# Human miR-1 Stimulates Metabolic and Thermogenic-Related Genes in Adipocytes

**DOI:** 10.3390/ijms26010276

**Published:** 2024-12-31

**Authors:** Ester Díez-Sainz, Fermín I. Milagro, Paula Aranaz, José I. Riezu-Boj, Pierre-Louis Batrow, Laura Contu, Nadine Gautier, Ez-Zoubir Amri, Isabelle Mothe-Satney, Silvia Lorente-Cebrián

**Affiliations:** 1 Department of Nutrition, Food Science and Physiology, and Center for Nutrition Research, Faculty of Pharmacy and Nutrition, University of Navarra, 31008 Pamplona, Spain; ediezsainz@alumni.unav.es (E.D.-S.); paranaz@unav.es (P.A.); jiriezu@unav.es (J.I.R.-B.); 2 Navarra Institute for Health Research (IdiSNA), 31008 Pamplona, Spain; 3 Centro de Investigación Biomédica en Red Fisiopatología de la Obesidad y Nutrición (CIBERobn), Instituto de Salud Carlos III, 28029 Madrid, Spain; 4 CNRS, Inserm, Institut de Biologie Valrose (iBV), Université Côte d’Azur, 06107 Nice, France; pierre-louis.batrow@univ-cotedazur.fr (P.-L.B.); laura.contu@univ-cotedazur.fr (L.C.); nadine.gautier@univ-cotedazur.fr (N.G.); ez-zoubir.amri@univ-cotedazur.fr (E.-Z.A.); isabelle.satney@univ-cotedazur.fr (I.M.-S.); 5 Department of Pharmacology, Physiology and Legal and Forensic Medicine, Faculty of Health and Sport Science, University of Zaragoza, 50009 Zaragoza, Spain; slorentec@unizar.es; 6 Instituto Agroalimentario de Aragón-IA2, Universidad de Zaragoza-Centro de Investigación y Tecnología Agroalimentaria (CITA), 50013 Zaragoza, Spain; 7 Aragón Health Research Institute (IIS-Aragon), 50009 Zaragoza, Spain

**Keywords:** brown adipocyte, white adipocyte, adipocyte browning, *PTK9*, *TWF1*, *UCP1*, miR-1, microRNA

## Abstract

MicroRNAs play a pivotal role in the regulation of adipose tissue function and have emerged as promising therapeutic candidates for the management of obesity and associated comorbidities. Among them, miR-1 could be a potential biomarker for metabolic diseases and contribute to metabolic homeostasis. However, thorough research is required to fully elucidate the impact of miR-1 on human adipocyte thermogenesis and metabolism. This study aimed to explore the effect of miR-1 on human adipocyte browning, a process whose activation has been linked to obesity protection and counteraction. Human multipotent adipose-derived stem cells, hMADS cells, were differentiated into white and brown-like adipocytes and transfected with miR-1 mimics for gene expression and western blotting analyses. miR-1 inhibited the expression of its previously validated target *PTK9*/*TWF1* and modulated the expression profile of key genes involved in thermogenesis and adipocyte browning (increased *UCP1* at mRNA and protein level, increased *CPT1M*, decreased *HIF3A*), adipocyte differentiation and metabolism (decreased *PLIN1*, *FASN*, *RXRA*, *PPARG*, *FABP4*, *MAPKAPK2*), as well as genes related to the cytoskeleton (decreased *ACTB*) and extracellular matrix (decreased *COL1A1*). These findings suggest that miR-1 can modulate the expression of adipocyte human genes associated with thermogenesis and metabolism, which could hold value for eventual therapeutic potential in obesity.

## 1. Introduction

Obesity is a chronic metabolic disease characterized by a positive energy balance that eventually leads to an excessive fat accumulation [1]. Obesity is considered an emerging epidemic that affects more than 1 billion people worldwide, and it is estimated that by 2030, up to 57.8% of the world adult population could be overweight or obese [2,3]. Obesity is a major health problem that is associated with the onset and/or progression of several comorbidities, including gastrointestinal disorders, cardiovascular diseases, type 2 diabetes, non-alcoholic fatty liver disease (NAFLD), mental disorders, and cancer, and is considered a risk factor for severe outcome of infectious diseases, such as COVID-19 or influenza [4,5,6,7,8,9,10,11].

Adipose tissue is a key metabolic and endocrine organ involved in the regulation of energy homeostasis that can be mainly divided into white adipose tissue (WAT), brown adipose tissue (BAT), and brite/beige adipose tissue [12,13,14,15]. WAT main function is energy storage, being specialized in lipid storage and mobilization [12,14,16]. Lipid storage and mobilization (lipolysis) are regulated by several proteins, such as FABP4 (Fatty Acid Binding Protein 4), PLIN1 (Perilipin 1), ATGL (Adipose Triglyceride Lipase), and HSL (Hormone-sensitive Lipase) [14,17,18]. The positive energy balance established during obesity results in fat accumulation in WAT, which leads to a dysfunctional and inflamed WAT that in turn disrupts global metabolic homeostasis, including the promotion of hyperglycemia, lipotoxicity, systemic insulin resistance, and liver steatosis [19,20,21]. On the other hand, the primary function of brown/brite adipocytes is energy expenditure and thermogenesis [12,16]. In adults, white adipocytes can undergo conversion into beige/brite adipocytes, which are similar to brown adipocytes, in a process called browning [12,13,16]. BAT and beige adipose tissue display higher activity and biosynthesis of mitochondria, which is accompanied by the enhancement of UCP-1 (Uncoupling Protein 1) expression [13,16]. UCP-1 expression is regulated by several proteins, such as CIDEA (Cell Death Inducing DFFA Like Effector A), PPARγ, and RXRα [22]. BAT activity is positively correlated with energy expenditure, and its prevalence is significantly lower in subjects with obesity [23,24]. Remarkably, BAT function has been associated with improvement of metabolic health in obesity and the decrease of liver fat accumulation, whereas impairment of BAT activity is associated with obesity progression [12,25,26]. Thus, therapeutic strategies aiming to enhance BAT activity and/or beige adipocyte number could be a useful tool to enhance energy expenditure, to restore energy balance disrupted during obesity, and to ameliorate metabolic diseases associated with obesity [12,16,26,27].

MicroRNAs (miRNAs) are small non-coding RNA molecules that act as post-transcriptional gene expression regulators through their binding by base complementarity to messenger RNAs (mRNAs), suppressing translation or promoting their degradation [28]. In animals, microRNAs are involved in the regulation of a plethora of biological processes, including metabolism, inflammation, cell proliferation, apoptosis, and differentiation [29,30,31]. Dysregulation of miRNAs expression has been associated with several human diseases, such as metabolic diseases, cardiovascular diseases, and cancer [29,30,31,32]. Notably, miRNAs play a key role in the regulation of adipose tissue gene expression, influencing tissue physiology, including adipogenesis, browning, lipogenesis, lipolysis, and adipokine secretion [33]. Indeed, miRNAs have emerged as novel biomarkers of adipose tissue function and obesity, standing out as promising therapeutic targets for the management of metabolic diseases [34,35]. The therapeutic use of miRNAs in obesity has been investigated in animal models, unveiling that the injection of miRNAs can decrease fat deposition, promote the restoration of glucose tolerance, ameliorate inflammation, and enhance WAT browning and thermogenesis [36,37,38]. In this context, miR-1 has been found downregulated during the development of obesity in mice and increased in individuals with pre-diabetes and was associated with important features of pre-diabetes, suggesting that it could be a biomarker for metabolic diseases [39,40]. Remarkably, increased miR-1 expression through its delivery in extracellular vesicles increased mice WAT lipolysis [41]. In addition, injection of miR-1 improved insulin sensitivity of obese mice through the enhancement of oxidative metabolism and mitochondrial content and the regulation of mitochondrial respiration [42]. Nevertheless, as far as we know, the role of miR-1 in human adipocyte metabolism and thermogenesis has not been explored yet. The aim of the present study was to evaluate the role of miR-1 on the browning process of human white adipocytes and to determine its potential impact on the expression of adipocyte genes involved in metabolism and thermogenesis.

## 2. Results

### 2.1. Direct Putative Targets of miR-1 Could Be Associated with the Modulation of Metabolic Biological Processes

**Figure 1:**
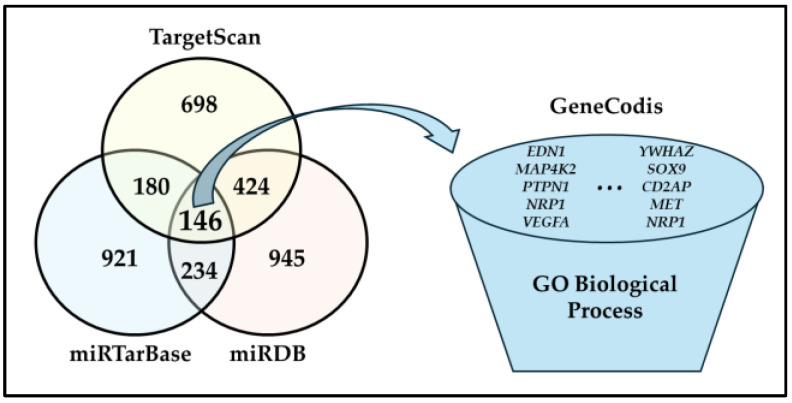
In silico identification of putative human target genes of miR-1. The miRNA-target prediction online tools TargetScan (version 8.0; https://www.targetscan.org/vert_80/), miRTarBase (https://awi.cuhk.edu.cn/~miRTarBase/miRTarBase_2025/php/index.php), and miRDB (https://mirdb.org/) were used to predict human targets of miR-1 (accessed on 5 September 2024). Target genes identified by the three prediction algorithms were used to perform a Gene Ontology enrichment analysis with GeneCodis (https://genecodis.genyo.es/; accessed on 5 September 2024). The Figure represents a schematic illustration of the bioinformatic workflow, and it shows the number of target genes identified by each prediction algorithm, as well as those common to more than one tool. Abbreviations: GO (Gene Ontology).

We selected miR-1 as candidate microRNA after conducting bibliographic analysis [39,40,41,42]. As a first preliminary approach to unravel the mechanism of action and target gene interactions by which miR-1 could promote adipocyte browning, we performed an in silico analysis to predict miR-1 putative human targets and biological functions. We identified potential human target genes of miR-1 by using TargetScan version 8.0 (https://www.targetscan.org/vert_80/) [43], miRTarBase (https://awi.cuhk.edu.cn/~miRTarBase/miRTarBase_2025/php/index.php) [44], and miRDB (https://mirdb.org/) (accessed on 5 September 2024) [45]. Bioinformatic analyses revealed that 698 (TargetScan), 921 (923 different transcripts; miRTarBase) and 945 (miRDB) genes were potential human targets of miR-1, of which 146 genes were common outputs to the three servers, including *PTK9*/*TWF1* (Figure 1).

To identify biological processes modulated by the set of 146 putative targets of miR-1, of which one (*ANKRD29*) was an unannotated input, we conducted Gene Ontology (GO) enrichment analysis by using GeneCodis (https://genecodis.genyo.es/; accessed on 5 September 2024). The biological processes that could be modulated by miR-1 putative targets were diverse, including pathways such as mRNA transcription (*DDX5*, *HIPK3*), angiogenesis (*YWHAZ*, *PARVA*, *FN1*, *HAND2*, *NRP1*, *VEGFA*, *PDCD10*, *WASF2*, *RNF213*) and clathrin-dependent endocytosis (*CLTC*, *PICALM*, *GAK*) (Supplementary Table S1). Notably, miR-1 predicted targets were enriched in biological processes that could have an impact on metabolism and/or adipocyte browning, such as positive regulation of JUN kinase activity (*EDN1*, *MAP4K2*, *PTPN1*) [46,47], VEGF-activated neuropilin signalling pathway (*NRP1*, *VEGFA*) [48,49], ERK1 and ERK2 cascade (*EDN1*, *YWHAZ*, *SOX9, CD2AP*), hepatocyte growth factor receptor signalling pathway (*MET*, *NRP1*) [50], and response to leptin (*EDN1*, *CCND1*) [51,52] (Supplementary Table S1).

### 2.2. Characterization of the In Vitro Model of White and Brown-like Adipocytes

We studied miR-1 impact on the expression profile of genes involved in key adipocyte processes, including thermogenesis, adipogenesis, and metabolism. We used multipotent adipose-derived stem (hMADS) cells isolated from human adipose tissue, differentiated into white adipocytes and transfected with miRNA mimics and then converted into brite adipocytes. After thorough bibliographic analysis, we decided to analyse in a more detailed manner the following genes, which are directly related to adipocyte phenotype: (1) lipolysis (*ATGL*, *HSL*, *PLIN1*, *ADRB3*), (2) lipid synthesis (*FASN*, *FABP4*), (3) lipid oxidation (*ACOX1*, *CPT1M*), (4) adipogenesis (*PPARG*, *RXRA*, *CIDEA*), (5) thermogenesis (*UCP1*, *CPT1M*, *CIDEA*), (6) cytoskeleton and extracellular matrix (*COL1A*, *ACTB*), and (7) hypoxia and intracellular signalling (*HIF3A*, *MAPKAPK2*). In addition, *PTK9/TWF1* gene expression was evaluated to determine the efficiency of the transfection procedure, since it is a validated direct target of miR-1 [53].

**Figure 2:**
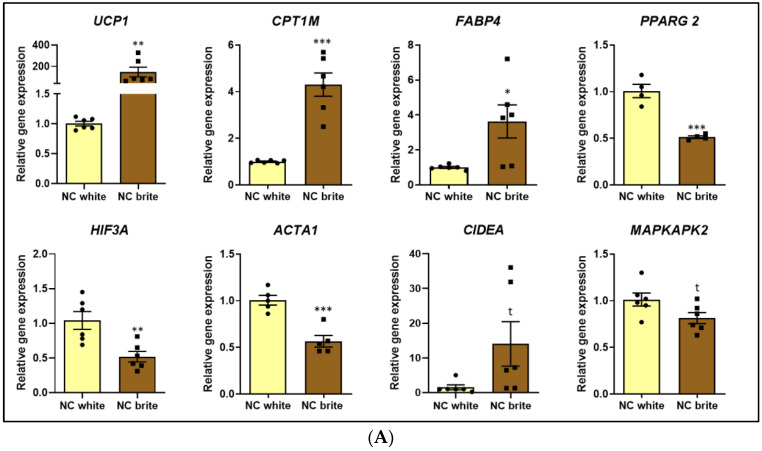

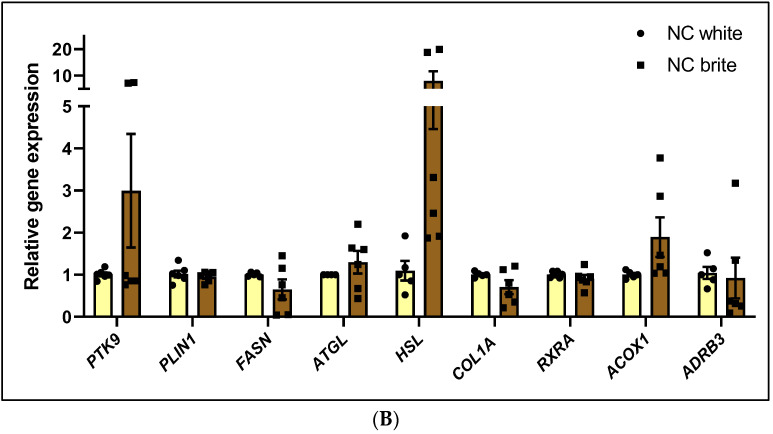
Conversion of white adipocytes into brown-like adipocytes. (**A**) mRNA expression of *UCP1*, *CPT1M*, *FABP4*, *PPARG 2*, *HIF3A*, *ACTA1*, *CIDEA*, *MAPKAPK2*. (**B**) mRNA expression of *PTK9*, *PLIN1*, *FASN*, *ATGL*, *HSL*, *COL1A*, *RXRA*, *ACOX1*, *ADRB3*. hMADS cells were subjected to differentiation at day 0, transfected at day 10–12 with 25 nM of a scramble sequence (negative control), and stimulated to undergo browning between day 14 and 18. mRNA expression was quantified at day 18 of differentiation by qPCR and normalized with the *36B4* housekeeping gene. The data represented are the relative gene expression mean (comparisons made with negative control white using the 2^−ΔΔCt^ method) ± standard error of the mean (SEM) (n = 4–6), and significance was determined with Student two-tailed *t*-test. *p*-value: * *p* < 0.05, ** *p* < 0.01, *** *p* < 0.001, t (CIDEA) = 0.0813, t (MAPKAPK2) = 0.0551. Abbreviations: *ACOX1* (Acyl-CoA Oxidase 1), *ACTB* (Actin Beta), *ADRB3* (Adrenoceptor Beta 3), *ATGL* (Adipose Triglyceride Lipase), *CIDEA* (Cell Death Inducing DFFA Like Effector A), *COL1A1* (Collagen Type I Alpha 1 Chain), *CPT1M* (Carnitine palmitoyltransferase I), *FABP4* (Fatty Acid Binding Protein 4), *FASN* (Fatty Acid Synthase), *HIF3A* (Hypoxia Inducible Factor 3 Subunit Alpha), *HSL* (Hormone-sensitive Lipase), *PLIN1* (Perilipin 1), *MAPKAPK2* (MAPK Activated Protein Kinase 2), NC (negative control), *PPARG* (Peroxisome Proliferator-Activated Receptor Gamma), *PTK9* (Protein Tyrosine Kinase 9), *RXRA* (Retinoid X Receptor Alpha), *UCP1* (Uncoupling Protein 1).

Comparison of the gene expression profile of non-transfected white and brown-like adipocytes has been previously described [54]. Nevertheless, since the aim of the study was to evaluate miR-1 impact upon synthetic mimics transfection, we analysed the conversion of white adipocytes into brown-like adipocytes by comparing the mRNA expression profile of scramble-sequence transfected white adipocytes (negative control white) versus scramble-sequence transfected brown-like adipocytes (negative control brite) (Figure 2) [55]. We made a special focus on browning markers, such as *UCP1* and *CTP1M*, which significantly increased, confirming the efficiency of the browning induction with rosiglitazone (Figure 2A). In addition, *FABP4* was overexpressed, while *ACTIN*, *HIF3A*, and *PPARG2* were downregulated after the browning stimulation (Figure 2A). Of note, *CIDEA* showed a trend towards the upregulation whereas *MAPKAPK2* showed a trend towards the downregulation in brite adipocytes (Figure 2A). The browning induction did not modify the expression of *ACOX1*, *ADRB3*, *ATGL*, *COL1A*, *FASN*, *HSL*, *PLIN1*, *PTK9*, and *RXRA* (Figure 2B).

### 2.3. MicroRNA miR-1 Modulates mRNA Expression of PTK9 Target and Genes Involved in Thermogenesis and Metabolism in Human Brown-like Adipocytes

**Figure 3:**
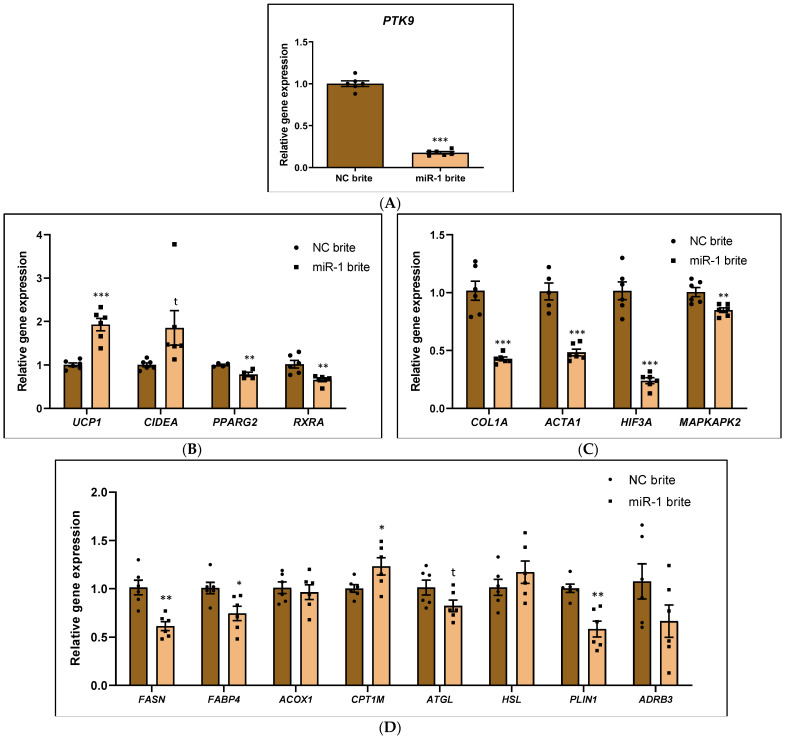
Effect of miR-1 on the gene expression profile of brown-like adipocytes. (**A**) mRNA expression of miR-1 putative target *PTK9*. (**B**) mRNA expression of genes involved in thermogenesis (*UCP1*, *CIDEA*) and adipogenesis (*PPARG*, *RXRA*). (**C**) mRNA expression of genes involved in extracellular matrix remodeling (*COL1A*, *ACTB*) and intracellular response (*HIF3A*, *MAPKAPK2*). (**D**) mRNA expression of genes involved in lipid metabolism and lipid turnover (FASN, FABP4, ATGL, HSL, PLIN1, ADRB3, ACOX1, CPT1M). hMADS were subjected to differentiation at day 0, transfected at day 10–12 with 25 nM of hsa-miR-1 or a scramble sequence (negative control), and stimulated to undergo browning between day 14 and 18. mRNA expression was quantified at day 18 of differentiation by qPCR and normalized with the *36B4* housekeeping. The data represented are the relative gene expression mean (comparisons made with negative control brite using the 2^−ΔΔCt^ method) ± standard error of the mean (SEM) (n = 4–6), and significance was determined with Student two-tailed *t*-test. *p*-value: * *p* < 0.05, ** *p* < 0.01, *** *p* < 0.001, t (*ATGL*) = 0.0793; t (*CIDEA*) = 0.0598. Abbreviations: *ACOX1* (Acyl-CoA Oxidase 1), *ACTB* (Actin Beta), *ADRB3* (Adrenoceptor Beta 3), *ATGL* (Adipose Triglyceride Lipase), *CIDEA* (Cell Death Inducing DFFA Like Effector A), *COL1A1* (Collagen Type I Alpha 1 Chain), *CPT1M* (Carnitine palmitoyltransferase I), *FABP4* (Fatty Acid Binding Protein 4), *FASN* (Fatty Acid Synthase), *HIF3A* (Hypoxia Inducible Factor 3 Subunit Alpha), *HSL* (Hormone-sensitive Lipase), *PLIN1* (Perilipin 1), *MAPKAPK2* (MAPK Activated Protein Kinase 2), NC (negative control), *PPARG* (Peroxisome Proliferator-Activated Receptor Gamma), *PTK9* (Protein Tyrosine Kinase 9), *RXRA* (Retinoid X Receptor Alpha), *UCP1* (Uncoupling Protein 1).

Comparison of *PTK9* mRNA expression between miR-1 transfected brite adipocytes and negative control brite adipocytes revealed that miR-1 markedly downregulated its predicted target (−82.50% ± 3.59; *p* < 0.001), which confirmed the efficiency of the transfection procedure (Figure 3A). Importantly, when this was compared with negative control brite adipocytes, miR-1-transfected adipocytes also showed reduced mRNA levels of the genes *FABP4* (−26.33% ± 9.46; *p* < 0.05), *PLIN1* (−42.17% ± 9.21; *p* < 0.01), *FASN* (−40.00% ± 8.85; *p* < 0.01), *PPARG2* (−21.75% ± 5.50; *p* < 0.01), *HIF3A* (−77.50% ± 8.12; *p* < 0.001), *ACTB* (−52.67% ± 7.30; *p* < 0.001), *COL1A* (−58.83% ± 8.45; *p* < 0.001), *RXRA* (−36.00% ± 9.59; *p* < 0.01) and *MAPKAPK2* (−15.67% ± 4.41; *p* < 0.01), along with a trend towards the decline of *ATGL* expression (−19.00% ± 9.73; t = 0.0793) (Figure 3B–D). Remarkably, miR-1 enhanced the mRNA expression of the brown markers *UCP1* (92.17% ± 14.86; *p* < 0.001) and *CPT1M* (22.83% ± 9.73; *p* < 0.05), which was accompanied by a tendency towards increases in *CIDEA* expression (84.83% ± 39.98; t = 0.0598) (Figure 3B,D). Of note, as compared with negative control brite adipocytes, miR-1 did not affect the expression of *ACOX1*, *ADRB3*, and *HSL* (Figure 3D). Data concerning lipid accumulation in the cells, measured by oil Red (ORO) staining, are shown in Supplementary Figure S1. Gene expression is in agreement with this observation.

### 2.4. MicroRNA miR-1 Increases UCP-1 Protein Expression but Does Not Affect Mitochondriogenesis in Human Brown-Like Adipocytes

**Figure 4:**
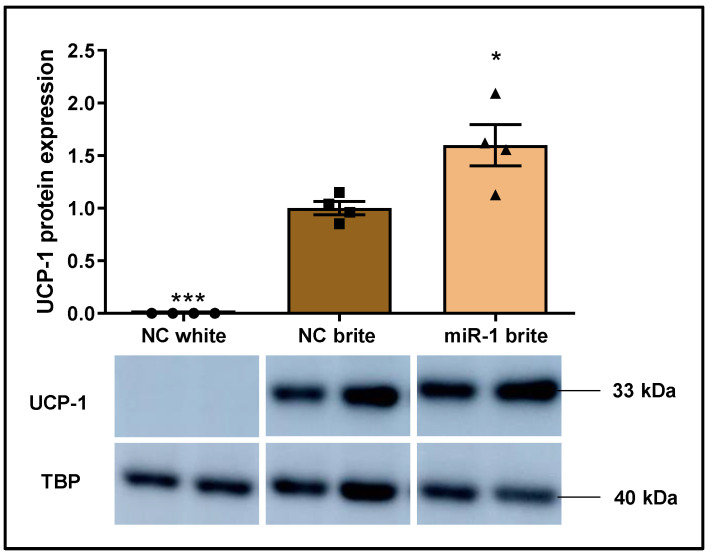
Effect of miR-1 on UCP-1 protein expression profile of brown-like adipocytes. hMADS cells were subjected to differentiation at day 0, transfected at day 10–12 with 25 nM of hsa-miR-1 or a scramble sequence (negative control), and stimulated to undergo browning between day 14 and 18. Western blot was performed at day 18 of differentiation to quantify UCP-1 protein levels, whose signal band intensity was analysed with ImageJ and normalized to TBP housekeeping. The Figure depicted representative images of western blot analysis and graph represented UCP-1 protein expression mean (comparison made with the negative control brite) ± standard error of the mean (SEM) (n = 4). Significance was determined with the Student two-tailed *t*-test when compared to NC brite. *p*-value: * *p* < 0.05, *** *p* < 0.001. Abbreviations: NC (negative control), *UCP1* (Uncoupling Protein 1).

We studied UCP-1 protein expression to determine the functional impact of miR-1 on human brite adipocytes thermogenesis, since UCP-1 regulation is a crucial event of the browning process [56]. UCP-1 protein expression was undetectable in white adipocytes but increased upon stimulation of white adipocytes conversion into brite adipocytes (Figure 4), being consistent with the mRNA data that supported the efficiency of the rosiglitazone-browning stimulation (Figure 2A). Remarkably, the upregulation of UCP-1 mRNA expression induced by miR-1 in brite adipocytes (Figure 3B) translated to an enhancement of UCP-1 protein levels, as compared to negative control brite adipocytes (59.79% ± 20.71; * *p* < 0.05) (Figure 4).

Along with UCP-1 increase, mitochondriogenesis enhancement is another important event during brown adipocyte conversion [54]. We estimated mitochondria biogenesis by quantifying mitochondrial DNA. Mitochondria DNA quantity was higher in negative control brite adipocytes than in negative control white adipocytes (40.28% ± 4.17; *p* < 0.05) (Supplementary Figure S2). This result suggests an increase of mitochondrial number after rosiglitazone treatment, providing robustness to gene and protein expression data that support the efficiency of the brown-like phenotype stimulation (Figure 2, Figure 4 and Supplementary Figure S2). However, the data suggest that miR-1 transfection did not affect mitochondriogenesis as compared to negative control brite adipocytes (Supplementary Figure S2).

## 3. Discussion

MiRNAs have emerged as key regulators of adipose tissue function, highlighting their therapeutic potential as targets and biomarkers for managing metabolic diseases [34,35]. Comprehensive evidence has suggested promising beneficial effects for miR-1 in restoring metabolic homeostasis during obesity [39,40,41,42]. In light of this evidence, and given the association between BAT activation and obesity alleviation [57,58], the present article ought to evaluate the impact of miR-1 on adipose browning process and metabolic expression profile in human adipocytes.

The in silico analysis, conducted to predict targets and biological functions of miR-1, revealed that miR-1 could potentially interact with genes involved in the response to leptin, which plays a crucial role in adipogenesis and adipocyte browning stimulation [51,52]. In line with our results, it has been demonstrated that miR-1 could reduce mice insulin resistance through AMPK activation [42], which is an important mediator of leptin effects [59]. Furthermore, the bioinformatic approach reported that other biological pathways associated with metabolism and/or adipocyte browning could be modulated by miR-1: the hepatocyte growth factor receptor signalling pathway, which has been linked to obesity and insulin resistance protection [50]; the regulation of JUN kinase activity, whose inactivation enhances energy expenditure and counteracts metabolic dysregulation in obesity [47]; and VEGF-activated neuropilin signalling pathway. Notably, VEGF could modulate brown adipose tissue maintenance and development [48], and in agreement with our results, reported evidence has shown that VEGFA is a direct target of miR-1 [60]. We hypothesized that miR-1 could modulate the metabolic gene expression profile of human adipocytes and promote adipocyte browning. This hypothesis relies on the in-silico analysis that reported the potential impact of miR-1 on the modulation of biological processes related to adipocyte browning and metabolism, as well as the biographic evidence supporting a role for miR-1 on the alleviation of metabolic disturbances during obesity [39,40,41,42]. This hypothesis was validated using human multipotent adipose-derived stem cells (hMADS) differentiated into white and brown-like adipocytes, an in vitro model previously used to evaluate the impact of miRNAs from plant and animal species on adipocyte differentiation, adipogenesis, metabolism, and browning [55,61,62]. hMADS originate from pediatric biopsies of subcutaneous adipose tissue. These cells exhibit at a clonal level a normal karyotype, self-renewal ability, the absence of tumorigenicity, are able to differentiate into various lineages, including adipocytes and osteoblasts, and can also support in vivo regenerative processes. The establishment and characterization of multipotency and self-renewal capacity of hMADS cells have been described previously [63]. In the experiments reported herein, hMADS-3 cells were used and came originally from the prepubic fat pad of a 4-month-old male. Cells were used between passages 14 and 25, corresponding from 35 to 100 population doublings, and all experiments have been performed at least three times using different cultures.

This work provides the first evidence of the impact of miR-1 on increasing *UCP1* gene and protein expression, the main driver of the browning process, involved in energy expenditure and thermogenesis [54,64]. However, this effect of miR-1 on UCP-1 expression seems to be mediated through an indirect modulation. We observed a tendency towards the increase of *CIDEA*, which correlates with previous evidence that CIDEA promotes UCP1 expression [22]. A remarkable effect induced by miR-1 was a great downregulation of *HIF3A* mRNA levels, a subunit of the hypoxia-inducible transcription factor whose inhibition has been associated with *UCP1* upregulation and the triggering of mice white adipocyte browning [65]. Importantly, our results are aligned with the counter-regulatory axis HIF3A-UCP1 as previously reported [65]. Notably, miR-1 also increased *CPT1M/CPT1B* gene expression, which is another marker of the browning process, also involved in the promotion of lipid oxidation [64,66,67]. Consistent with these results, it has been suggested that miR-1 could increase muscle oxidative metabolism [42]. By contrast, miR-1 downregulated *PLIN1*, which has a crucial role on lipid metabolism homeostasis and its silencing, has been associated with lipolysis enhancement in vitro [17,68,69]. In agreement with these results, other studies have reported that increased miR-1 expression enhances WAT lipolysis [41].

miR-1 also decreased the gene expression of *PPARG*, a master regulator of lipid metabolism and adipogenesis [70]. *PPARG* inhibition was accompanied by a decrease of mRNA levels of the pivotal lipogenic enzyme *FASN*, which agrees with the fact that FASN is a downstream target of PPARγ [71,72,73]. PPARγ heterodimerizes with RXRα to regulate glucose and lipid metabolism and adipogenesis [74,75]. RXRα is a retinoid X receptor whose mRNA levels decreased after miR-1 transfection. miR-1 has been also related to the suppression of lipogenesis in hepatocytes through the inhibition of LXRα, which forms heterodimers with RXRα, and the eventually decrease of lipogenic genes like FASN [76], in agreement with our results.

Overall, our results suggest that miR-1 could modulate the expression profile of key genes involved in adipocyte thermogenesis and metabolism, towards a potential promotion of energy expenditure, lipid oxidation, and lipolysis (through *UCP1* and *CPT1M* upregulation and *HIF3A* and *PLIN1* downregulation), as well as an inhibition of lipogenesis (through *PPARG*, *RXRA*, and *FASN* downregulation). Interestingly, specific inhibition of these genes did not translate to functionally relevant changes on lipid accumulation as regards of ORO staining results align to these observations. Stimulation of lipid catabolism (lipolysis and lipid oxidation) and inhibition of anabolic pathways (like fatty acid synthesis) by miR-1 suggests a strong regulatory role of this miRNA on activating adipocyte browning.

Notably, miR-1 downregulated the expression of the predicted target gene *PTK9/TWF1*, which has been validated in previous studies [53]. Indeed, it has been demonstrated that overexpression of miR-1 reduces the luciferase activity of the reporter containing the 3’UTR of PTK9/TWF1 mRNA. *PTK9* encodes for the twinfilin-1 protein, linked to cytoskeletal remodeling and myogenic differentiation [77]. Cytoskeleton remodeling is essential to promote differentiation of preadipocytes into mature adipocytes and adipogenesis [78]. In this context, miR-1 modulated the expression of cytoskeleton and extracellular matrix remodeling genes (*ACTB* and *COL1A*). However, a direct association between *PTK9* and adipogenesis and/or adipocyte browning has not been revealed yet, and we acknowledge that miR-1 might affect adipocyte metabolism and thermogenesis gene expression profile through the direct modulation of additional genes. Indeed, some of the miR-1 predicted genes depicted in Supplementary Table S1 had been linked to the regulation of thermogenesis and/or browning. Of particular interest are PTPN1 (coding for PTP1B) and VEGFA, which have been previously related to the physiology of adipose tissue. Protein-tyrosine phosphatase 1B (PTP1B, encoded by PTPN1) is a physiological regulator of glucose homeostasis and adiposity and thus, has been considered a drug target for the treatment of obesity and diabetes [79]. Indeed, deletion of PTP1B enhanced insulin and leptin signaling in hypothalamus and increased WAT browning and energy expenditure [80]. Although this effect was observed in vivo, these results strongly support that miR-1-induced inhibition of PTPN1 might promote WAT browning eventually at cell specific (adipocyte) level [80,81,82,83]. On the other hand, VEGFA has been also identified as a miRNA-regulated target involved in WAT function, particularly on promotion of brown adipose tissue thermogenic programming [38,84]. For example, the upregulation of miR-206 leads to decreased expression of VEGFA, critical for BAT activation [85]. Therefore, further studies should elucidate the specific mechanisms and temporal dynamics involved in the interaction between the genes regulated by miR-1, including the in vitro identification of the direct target genes that could trigger the miR-1 effects on human adipocytes.

The gene expression changes induced by miR-1 suggests a functional impact on brite adipocyte thermogenesis: miR-1 upregulated UCP-1 protein levels, which correlated with the increase of *UCP1* gene expression. These results suggest that miR-1 might have an impact on thermogenesis since *UCP1* transcriptional regulation is a crucial event for the acquisition of thermogenic properties during adipocyte browning [56,86]. Indeed, previous studies suggest that therapeutic strategies aimed at increasing *UCP1* expression, using synthetic and natural bioactive compounds, could be an effective approach to promote adipocyte browning and eventually combat metabolic diseases such as obesity [12,87,88,89,90]. However, miR-1 did not exert an impact of mitochondriogenesis, which is associated with adipocyte browning [91,92]. By contrast, it has been reported that miR-1 increases mitochondria content and regulates mitochondria respiration in mice skeletal muscle [42]. It could be speculated that miR-1 effects on mitochondriogenesis might be a medium-long term effect or that in vivo regulatory pathways might be necessary to eventually achieve these actions.

Substantial evidence has revealed the outstanding role of miRNAs on the regulation of beige and brown fat development and function, including the modulation of brown adipogenesis, thus highlighting their role as a novel class of therapeutic targets against metabolic diseases [93]. Our results are supported by previous studies where, for instance, miR-889-3p, miR-22, and miR-133a regulate thermogenesis and promote the browning process [94,95,96]. We have reported, for the first time, the effect of miR-1 on regulating metabolic and thermogenic genes in human brown-like adipocytes. miR-1 expression is reduced in adipose tissue during mice obesity onset [39]. Collectively, these findings highlight that miR-1 could be a relevant biomarker for metabolic diseases, including obesity [39,40], and suggest its promising therapeutic potential. miR-1-based treatments could be considered to restore metabolic homeostasis [41,42], and to enhance energy expenditure and adipocyte browning.

This study has several limitations that should be considered with caution: (1) Further in-depth studies will be necessary to fully determine the metabolic and thermogenic pathways and interactions modulated by miR-1, as well as the direct targets of miR-1 underlying the effect of this miRNA on human adipocytes. RNA sequencing analysis could provide deeper insights into the miRNA-target gene interactions; for example, the evaluation of oxygen consumption by the transfected cells might be studied by using Seahorse or Mito-OCR assay. (2) Given that this study focused on investigating miR-1 effects of hMADS cells, our findings warrant further validation in additional human cells lines, such as primary white subcutaneous adipocytes (PHWSC), primary isolated adipose stromal cells (ASCs), and/or SGBS (Simpson–Golabi–Behmel Syndrome) cells [97,98]. (3) Transfections were conducted to assess miR-1 effects in adipocytes, as it is a widely used and effective approach for investigating the impact of miRNAs [62,99,100]. However, future research could focus on other approaches, such as the encapsulation in extracellular vesicles, that could provide more physiological environments [101,102]. (4) Comprehensive functional assays using animal models will be required to evaluate the impact of miR-1 on adipocyte metabolism and browning and to determine their therapeutic potential. Given the possibility of genetic variations between humans and other animal species, it would be crucial to thoughtfully perform accurate bioinformatic predictions of miR-1 targets and to choose an appropriate model to fully characterize miR-1 function in vivo. While acknowledging these limitations, our study underscores a promising role for miR-1 on the modulation of thermogenesis and metabolism in human adipocytes, laying the groundwork for additional research into the therapeutic potential of miR-1 to maintain metabolic homeostasis and promote adipocyte browning.

## 4. Materials and Methods

### 4.1. Target Prediction In Silico Analysis

We performed the prediction of potential human target genes of hsa-miR-1 with publicly available databases: TargetScan version 8.0 (https://www.targetscan.org/vert_80/) [43], miRTarBase (https://awi.cuhk.edu.cn/~miRTarBase/miRTarBase_2025/php/index.php) [44], and miRDB (https://mirdb.org/) (accessed on 5 September 2024), applying the default parameters with the term “hsa-miR-1”. Aiming at selecting the most accurate miR-1-mRNA interactions, we considered for further analysis only common target genes to the three miRNA-target-prediction algorithms. We used this set of miR-1 putative human genes to conduct GO enrichment analysis with the annotation “GO Biological Process” of GeneCodis (https://genecodis.genyo.es/; accessed on 5 September 2024) [103]. We considered as enriched biological pathways those with an adjusted *p*-value < 0.05.

### 4.2. Cell Culture

The characterization of the human multipotent adipose-derived stem (hMADS) cells has been detailed in previous studies [63,104]. We cultured hMADS cells in low-glucose (1 g/L) DMEM with L-GlutaMAX (Gibco. Thermo Fisher Scientific Inc., Waltham, MA, USA), 1% penicillin-streptomycin solution (P/S; Gibco. Thermo Fisher Scientific Inc.), 10% fetal bovine serum (FBS; Eurobio, Les Ulis, France) and 15 mM HEPES (4-(2-hydroxyethyl)-1-piperazineethanesulfonic acid; Gibco. Thermo Fisher Scientific Inc.) and maintained at 37 °C and 5% CO_2_. To promote the proliferation of hMADS cells, we added 2.5 ng/mL of the mitogenic factor hFGF2 (human fibroblast growth factor 2; Peprotech. Thermo Fisher Scientific Inc.) to the medium before each use [105].

We seeded hMADS cells in 12 well-plates (25,000 cells per well) for gene expression analyses and in six-well plates (50,000 cells per well) for western blotting and mitochondrial DNA quantification analysis. We cultured the cells in low-glucose (1 g/L) DMEM with L-GlutaMAX, supplemented with 1% P/S, 10% FBS, 15 mM HEPES, and 2.5 ng/mL hFGF2 freshly added prior to each use. We removed hFGF2 to stop cell cycle after reaching confluence. We induced the differentiation of hMADS cells into adipocytes from 2-day post-confluence (designated as day 0 of the differentiation process) to day 18, using a medium consisting of low-glucose (1 g/L) DMEM with L-GlutaMAX and Ham’s F12 with L-glutamine (Gibco. Thermo Fisher Scientific Inc.) (1:1 ratio), with 1% P/S, 15 mM HEPES, 10 µg/mL transferrin (Tf; Sigma-Aldrich, San Luis, MO, USA), 10 nM insulin (Invitrogen. Thermo Fisher Scientific Inc.), and 0.2 nM triiodothyronine (T3; Sigma-Aldrich). In addition, we added 500 µM isobutyl-methylxanthine (IBMX; Sigma-Aldrich) and 1 µM dexamethasone (Dex; Sigma-Aldrich) from day 0 to day 4. To promote the differentiation of white adipocytes, we treated the cells with 100 nM rosiglitazone (Rosi; Cayman Chemical Company, Ann Arbor, MI, USA), which is a PPARγ agonist, from day 2 to day 9 [54,64]. We converted white adipocytes in brown-like adipocytes with 100 nM Rosi treatment for 96 h, from day 14 to day 18 [54,64]. Of note, Dex, IBMX, insulin, Rosi, T3, and Tf were freshly added before each use and medium was refreshed every other day. Oil Red O staining was performed as described previously [55,63] and quantified by measuring absorbance at 490 nm in a spectrophotometer.

### 4.3. miRNA Mimic Transfection

At day 10–12 of the differentiation process hMADs display a typical mature-like adipocyte phenotype and had been functionally characterized previously [63,104]. At day 10–12 post-differentiation, we transfected hMADS cells with Lipofectamine RNAiMAX Reagent and 25 nM hsa-miR-1 mimic (mirVana™ miRNA Mimic, miR-1 Positive Control; Thermo Fisher Scientific Inc.). We stablished the negative control by performing transfection with 25 nM scramble sequence (mirVana™ miRNA Mimic, Negative Control #1). Immediately before transfections, we replaced the cell culture medium with low-glucose (1 g/L) DMEM with L-GlutaMAX and Ham’s F12 with L-glutamine (1:1 ratio) supplemented with 1% P/S, 15 mM HEPES, 10 µg/mL Tf, 10 nM insulin, and 0.2 nM T3 (450 µL per well for 12 well/plates and 900 µL per well for six well/plates). Then, we diluted mirVana™ miRNA Mimics and Lipofectamine RNAiMAX Reagent using Opti-MEM I Reduced Serum Medium (Gibco. Thermo Fisher Scientific Inc.) (1:1 ratio), followed by 10 min room temperature (RT) incubation. We transfected for 24 h with adding the following volumes of miRNA mimic-lipofectamine complexes, 150 µL (for 12-well plates) and 300 µL (for six-well plates), and lipofectamine volumes of 4.5 µL (for 12 well-plates in 600 µL volume) and 9 µL (for six-well plates in 1200 µL volume). After 24 h of transfection, we changed the medium and hMADS continued differentiating until day 18.

### 4.4. Experimental Design for In Vitro Treatments

hMADs were grown and cultured as described (see Section 4.2) and differentiated into adipocytes from day 2 post-confluence up to day 18. Adipocyte differentiation was induced by incubating cells in the presence of a hormonal cocktail containing 10 µg/mL transferrin (Tf), 10 nM insulin, and 0.2 nM triiodothyronine (T3) throughout the whole differentiation process. Composition of this hormonal cocktail was variable along the differentiation procedure: (a) from day 0 to day 4, cells were incubated additionally with 500 µM isobutyl-methylxanthine (IBMX) and 1 µM dexamethasone (Dex), (b) to promote white adipocytes differentiation, cells were treated with 100 nM rosiglitazone (Rosi) from day 2 to day 9, (c) conversion of white adipocytes to brown-like adipocytes, cells were treated with 100 nM rosiglitazone for 96 h, from day 14 to 18. In the absence of rosiglitazone, adipocytes continued differentiation to typical white adipocytes. Media was changed every other day and additives (Dex, IBMX, insulin, Rosi, T3, Tf) were freshly added before medium change.

At day 10–12 of the differentiation process hMADs display a typical mature-like adipocyte phenotype (lipid accumulation and a round-like shape) had been functionally characterized previously [63,104]. At day 10–12 post-differentiation, we transfected hMADS cells with Lipofectamine RNAiMAX Reagent and 25 nM hsa-miR-1 mimic and negative control (scramble sequence) (mirVana™ miRNA Mimic, miR-1 Positive Control and mirVana™ miRNA Mimic, Negative Control #1; Thermo Fisher Scientific Inc.). This transfection method consisted of delivering lipofectamine-mimic complexes to cells previously seeded and attached to cell culture plates. Just prior to transfection, hMADS cell medium was freshly changed to DMEM low glucose (1 g/L) L-GlutaMAX/Ham’s F12 with L-glutamine supplemented with 15 mM HEPES, 1% P/S, 10 nM insulin, 10 µg/mL Tf and 0.2 nM T3 (450 uL for 12-well plates and 900 uL for six-well plate formats). (1) miRNA mimics and lipofectamine were diluted in Opti-MEM I Reduced Serum Medium (Gibco), mixed in a 1:1 ratio, and incubated for 10 min at room temperature. (2) miRNA mimic-lipofectamine complexes were added to each well (transfection) for 24 h. Following volumes of miRNA mimic-lipofectamine complexes were added (Supplementary Table S5): 150 µL (for 12-well plates) and 300 µL (for six-well plates), and lipofectamine volumes of 4.5 µL (for 12 well-plates in 600 µL volume) and 9 µL (for six-well plates in 1200 µL volume). After 24 h of transfection, media was changed and replaced with new fresh media, and cells continued the differentiation process until day 18. At this point, cells were collected for gene expression, Western Blot (WB), Oil Red O, and/or mitochondrial DNA quantification assays.

**Figure 5:**
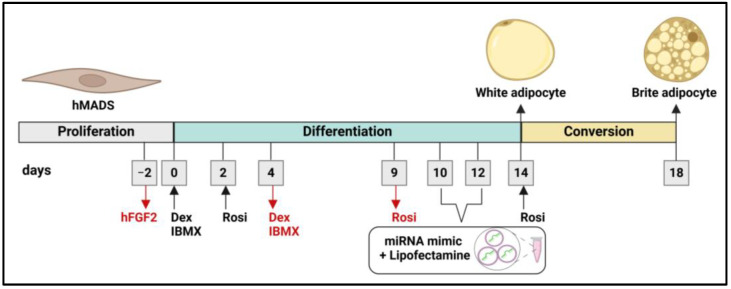
Differentiation of hMADS cells into white and brown-like adipocytes and transfection with hsa-miRNA mimics (miR-1 and Negative Control) at 25 nM. Abbreviations: Dex (Dexamethasone); Rosi (Rosiglitazone). Black and red arrows/text denote for addition or removal, respectively, of indicated medium components at specific days of differentiation. Image created using BioRender.

Experimental design consisting of adipocyte differentiation and transfection procedures are depicted in Figure 5.

### 4.5. RNA Isolation and Gene Expression Analysis

We studied gene expression analysis by quantifying mRNA levels of hMADS cells that were induced to differentiate for 18 days into white and brown-like adipocytes and underwent miRNA mimic transfection at day 10–12.

We scraped hMADS cells off the plate in TRI-Reagent (Molecular Research Center Inc., Cincinnati, OH, USA) and extracted the RNA following manufacturer’s protocol. We assessed RNA concentration and purity (260/280 ratio) in a NanoDrop 2000 (Thermo Fisher Scientific Inc.). Additionally, we confirmed RNA quality by electrophoresis analysis, staining RNA with GelRed Nucleic Acid Gel Stain (Biotium Inc., Fremont, CA, USA) mixed with BlueJuice Gel Loading Buffer (Invitrogen. Thermo Fisher Scientific Inc.).

We carried out RNA reverse-transcription following the manufacturer’s instructions. Briefly, we treated 1 µg RNA with RNAsin (Promega, Madison, WI, USA), RQ1 RNase-Free DNAse (Promega), and Reaction Buffer (M-MLV-RT kit; Promega). We incubated the mix at 37 °C for 15 min, 75 °C for 5 min, and 4 °C for 3 min, and incubated with random primers (Roche, Basel, Switzerland) at 70 °C for 5 min. Then, we incubated the samples at 37 °C for 60 min with M-MLV-RT and reaction buffer (M-MLV-RT kit; Promega) and dNTP Mix (Set of dATP, dCTP, dGTP, dTTP; Promega). We performed all the reactions in a Bioer GeneExplorer Thermal Cycler (Bioer Technology, Hangzhou, China).

We used cDNA (diluted 1/5) to perform quantitative PCR (qPCR) analysis, with primers designed with Primer Express Software (PerkinElmer and Analytical Sciences, Boston, MA, USA; http://www.perkinelmer.com, accessed on 26 December 2024) (Supplementary Table S2), primers predesigned from Integrated DNA Technologies (IDT; Coralville, IA, USA) (Supplementary Table S3), and ONEGreen FAST qPCR Premix (Ozyme, Saint-Cyr-l’École, France). We conducted the qPCR reactions on a StepOnePlus Real-Time PCR System (Applied Biosystems. Thermo Fisher Scientific Inc.) with cycling parameters of 95 °C for 30 s; 40 cycles at 95 °C for 5 s and 60 °C for 34 s; a melt curve stage of 95 °C for 15 s, 60 °C 1 min, 95 °C 15 s. We used the Cq values of the housekeeping control gene *36B4* (Ribosomal Protein Lateral Stalk Subunit P0, *RPLP0*) to normalize gene expression levels (∆Cq) [106]. We applied the 2^−∆∆Cq^ method to calculate relative gene expression levels, comparing the results with the ∆Cq values of the negative control (hMADS cells transfected with a scramble sequence) [107,108].

### 4.6. Protein Isolation and Western Blotting

We analysed protein expression in hMADS cells that underwent differentiation into white and brown-like adipocytes for 18 days and miRNA mimic transfection at day 10–12.

For protein isolation, we washed the cells with cold PBS (4 °C), scrapped off in protein lysis buffer (100 mM NaCl, 1 mM EDTA, 25 mM Tris-Cl (pH 7.4), 0.5% Nonidet P40, 1X protease inhibitor cocktail (Roche Life Sciences, Indianapolis, IN, USA), 0.5% Triton X-100), and sonicated (50–10 s, three times) them with a Sonifier ultrasonic homogenizer (Branson, Brookfield, CT, USA). We centrifuged the lysates at 14,000× *g*, 4 °C for 10 min to collect the supernatant. We quantified protein concentration with Pierce BCA Protein Assay kit (Thermo Fisher Scientific Inc.), measuring absorbance at 560 nm using an iMark™ Microplate Absorbance Reader (Bio-Rad, Hercules, CA, USA).

For Western Blotting analysis, we prepared the protein samples by diluting them to a final amount of 40 µg, in lysis buffer with 1% loading buffer Laemmli (0.01% bromophenol blue, 10% glycerol, 8% Sodium dodecyl-sulphate, 250 mM Tris-HCl pH 6.8), and 0.1 M DTT (dithiothreitol; Thermo Fisher Scientific Inc.), followed by heating at 95 °C for 5 min. We performed SDS-PAGE (Sodium dodecyl-sulphate polyacrylamide gel electrophoresis) in 11% gradients gels, running samples at 70 V for 30 min and 120 V for 2 h, using a power supply Hoefer EPS 2A200 (Hoefer Inc., Holliston, MA, USA). After electrophoresis, we transferred the proteins from gels into polyvinylidene fluoride (PVDF) membranes at 110 V, 200 mA for 1.5 h at 4 °C in a liquid transfer system Mini Trans-Blot^®^ Cell (Bio-Rad). We blocked the membranes with 5% skimmed milk diluted in TBST (Tris Buffered Saline and 0.05% tween) for 1 and RT. After TBST washing, we incubated the membranes overnight (ON) at 4 °C with the following rabbit primary antibodies diluted 1:1000: anti-TBP (anti-TATA-box binding protein; #44059, Cell Signaling Technology, Danvers, MA, USA) and anti-UCP1 (#Ab10983, Abcam, Waltham, MA, USA). In order to detect primary antibodies, after washing membranes with TBST, we incubated HRP (Horseradish peroxidase)-conjugated anti-rabbit antibody (Promega) at 1:10,000 dilution for 1 h at RT. We washed the membranes with TBST and visualized the HRP luminescence signal using Clarity Western ECL (enhanced chemiluminescence) Substrate (Bio-Rad) in an Amersham Imager 600 (GE Healthcare, Chicago, IL, USA). We quantified the band signal intensity with Image J software (https://imagej.net/ij/) and normalized UCP-1 data with those of the housekeeping TBP. We calculated the relative protein concentration (%) by comparing the results with the negative control.

### 4.7. DNA Isolation and Mitochondrial DNA Quantification Analysis

Mitochondriogenesis is a process linked to adipocyte browning [91,92]. We evaluated this process in hMADS cells differentiated for 18 cells into white and brown-like adipocytes and transfected at day 10–12. We assessed mitochondrial DNA levels, as a correlate with mitochondria biogenesis, by quantifying the mitochondrial gene *NADHdS1* (NADH dehydrogenase Subunit 1), whose expression was normalized with the *LPL* (Lipoprotein Lipase) gene [92,109].

We purified the DNA with PureLink Genomic DNA Mini Kit (Invitrogen. Thermo Fisher Scientific Inc.), according to manufacturer’s instructions. We assessed DNA quality and quantity with NanoDrop 2000 (Thermo Fisher Scientific Inc.). We conducted qPCR with DNA (2 ng), primers designed using Primer Express Software (PerkinElmer and Analytical Sciences, https://www.thermofisher.cn/cn/zh/home/technical-resources/software-downloads/primer-express-software-download.html) (Supplementary Table S4), and ONEGreen FAST qPCR Premix (Ozyme), in a StepOnePlus Real-Time PCR System (Applied Biosystems. Thermo Fisher Scientific Inc.), with the following parameters: 95 °C 5 min; 40 cycles at 95 °C 10 s and 60 °C 20 s; a melt curve stage of 95 °C 15 s, 60 °C 1 min, 95 °C 15 s. We used *LPL* mRNA levels to normalize *NADHdS1* gene expression, and applied the 2^−∆∆Cq^ method to calculate relative gene expression, in comparison to the negative control [107,108].

### 4.8. Statistical Analysis

We conducted the statistical analyses with the Student’s *t*-test (two tailed) in GraphPad Prism 6.0 for Windows (GraphPad Software Inc., La Jolla, CA, USA) to establish comparison between negative control white adipocytes vs. negative control brown-like adipocytes, and miR-1 brown-like adipocytes vs. negative control brown-like adipocytes. *p*-value < 0.05 was considered statistically significant.

## 5. Conclusions

The present study reveals that miR-1 modulate the expression of genes involved in thermogenesis and metabolism in human brown-like adipocytes, thereby enhancing the protein-expression levels of the key biomarker of the browning process UCP-1. Our results suggest a promising role for miR-1 on adipocyte metabolism regulation and browning stimulation, which could entail relevance for the alleviation of obesity and its comorbidities.

## Data Availability

The raw data supporting the conclusions of this article will be made available by the authors on request.

## Supplementary Materials

The following supporting information can be downloaded at: https://www.mdpi.com/article/10.3390/ijms26010276/s1.
