# Supplementary material for: Human miR-1 Stimulates Metabolic and Thermogenic-Related Genes in Adipocytes
Source: Int J Mol Sci. 2024 Dec 31;26(1):276. doi: 10.3390/ijms26010276 (PMC11720367; doi:10.3390/ijms26010276)
Supplement: Supplementary file 1 [file ijms-26-00276-s001.zip › ijms-3318398-supplementary.docx]

**Supplementary material**

**Supplementary Table 1.** Gene ontology (GO) enrichment analyses of putative human target genes of miR-1.

| Biological process | Relative enrichment | Adjusted *p*-value | Genes |
| --- | --- | --- | --- |
| Coronary artery morphogenesis (GO:0060982) | 55.41 | 0.0007 | *HAND2, NRP1, VEGFA, ARID2* |
| Positive regulation of mesenchymal stem cell differentiation (GO:2000741) | 93.50 | 0.0015 | *SOX6, SOX9, SOX5* |
| Positive regulation of cartilage development (GO:0061036) | 22.00 | 0.0284 |  |
| Positive regulation of chondrocyte differentiation (GO:0032332) | 19.68 | 0.0304 |  |
| Cartilage condensation (GO:0001502) | 19.68 | 0.0304 |  |
| Sprouting angiogenesis (GO:0002040) | 17.81 | 0.0042 | *PARVA, NRP1, VEGFA, THBS1, RNF213* |
| Positive regulation of endothelial cell migration (GO:0010595) | 11.51 | 0.0051 | *EDN1, ETS1, NRP1, VEGFA, THBS1, FOXP1* |
| Positive regulation of cardiac muscle hypertrophy (GO:0010613) | 20.78 | 0.0099 | *EDN1, IGF1, HAND2, TWF1* |
| Negative regulation of apoptotic process (GO:0043066) | 3.58 | 0.0099 | *YWHAZ, HSP90B1, IGF1, VEGFA, GOLPH3, HIGD1A, SOX9, CHST11, THBS1, PDCD10, HIPK3, ARF4, HSPD1, AXL* |
| VEGF-activated neuropilin signaling pathway (GO:0038190) | 124.66 | 0.0139 | *NRP1, VEGFA* |
| Positive regulation of axon extension involved in axon guidance (GO:0048842) | 35.62 | 0.0443 |  |
| Osteoblast differentiation (GO:0001649) | 6.82 | 0.0148 | *HNRNPU, CLTC, HAND2, ADAR, H3F3B, SNAI2, RRBP1* |
| Negative regulation of neuron apoptotic process (GO:0043524) | 6.15 | 0.0252 | *CCND1, BDNF, NRP1, KRAS, AXL, CD2AP, KDM2B* |
| Cartilage development (GO:0051216) | 9.44 | 0.0264 | *EDN1, SOX6, SOX9, CHST11, SOX5* |
| Regulation of axon extension involved in axon guidance (GO:0048841) | 83.11 | 0.0264 | *PLXNA4, NRP1* |
| Trigeminal nerve structural organization (GO:0021637) | 41.55 | 0.0410 |  |
| Branchiomotor neuron axon guidance (GO:0021785) | 35.62 | 0.0443 |  |
| Synaptic vesicle lumen acidification (GO:0097401) | 22.00 | 0.0284 | *ATP6V1A, ATP6V1B2, CLCN3* |
| Negative regulation of epithelial cell differentiation (GO:0030857) | 22.00 | 0.0284 | *CCND1, KRAS, SOX9* |
| Clathrin coat assembly (GO:0048268) | 22.00 | 0.0284 | *CLTC, PICALM, GAK* |
| Clathrin-dependent endocytosis (GO:0072583) | 13.36 | 0.0464 |  |
| Sympathetic nervous system development (GO:0048485) | 22.00 | 0.0284 | *PLXNA4, HAND2, NRP1* |
| Angiogenesis (GO:0001525) | 4.35 | 0.0284 | *YWHAZ, PARVA, FN1, HAND2, NRP1, VEGFA, PDCD10, WASF2, RNF213* |
| Clathrin coat disassembly (GO:0072318) | 62.33 | 0.0289 | *CLTC, GAK* |
| mRNA transcription (GO:0009299) | 62.33 | 0.0289 | *DDX5, HIPK3* |
| Sympathetic neuron axon guidance (GO:0097492) | 62.33 | 0.0289 | *EDN1, PLXNA4* |
| Substantia nigra development (GO:0021762) | 11.08 | 0.0304 | *YWHAQ, CALM1, ACTB, G6PD* |
| Neural crest cell development (GO:0014032) | 17.81 | 0.0369 | *EDN1, SNAI2, SOX9* |
| Cartilage morphogenesis (GO:0060536) | 49.86 | 0.0369 | *HAND2, SNAI2* |
| Golgi reassembly (GO:0090168) | 49.86 | 0.0369 | *YWHAZ, PDCD10* |
| Establishment of Golgi localization (GO:0051683) | 31.17 | 0.0498 |  |
| Intracellular protein transport (GO:0006886) | 3.94 | 0.0410 | *ARF3, AP3D1, CLTC, AP1S1, ARCN1, STX6, ARF4, IPO8* |
| Retrograde vesicle-mediated transport, Golgi to endoplasmic reticulum (GO:0006890) | 9.78 | 0.0410 | *ARF3, GOLPH3, ARCN1, ARF4* |
| Negative regulation of protein localization to plasma membrane (GO:1903077) | 14.96 | 0.0410 | *CLTC, PPP2R5A, PICALM* |
| Alternative mRNA splicing, via spliceosome (GO:0000380) | 15.58 | 0.0410 | *DDX5, HNRNPU, SRSF9* |
| Positive regulation of smooth muscle cell proliferation (GO:0048661) | 9.23 | 0.0410 | *EDN1, IGF1, THBS1, FOXP1* |
| ERK1 and ERK2 cascade (GO:0070371) | 9.59 | 0.0410 | *EDN1, YWHAZ, SOX9, CD2AP* |
| Biological process involved in interaction with symbiont (GO:0051702) | 41.55 | 0.0410 | *FN1, HSPD1* |
| Neural crest cell migration involved in autonomic nervous system development (GO:1901166) | 41.55 | 0.0410 | *FN1, NRP1* |
| Regulation of alternative mRNA splicing, via spliceosome (GO:0000381) | 9.07 | 0.0410 | *HNRNPA1, DDX5, TRA2B, HNRNPU* |
| Regulation of mitotic spindle assembly (GO:1901673) | 15.58 | 0.0410 | *HNRNPU, EML3, CCSAP* |
| Epithelial to mesenchymal transition (GO:0001837) | 9.07 | 0.0410 | *IGF1, DDX5, SNAI2, SOX9* |
| Amyloid-beta clearance by transcytosis (GO:0150093) | 35.62 | 0.0443 | *CLTC, PICALM* |
| Noradrenergic neuron differentiation (GO:0003357) | 35.62 | 0.0443 | *EDN1, HAND2* |
| Cardiac neural crest cell migration involved in outflow tract morphogenesis (GO:0003253) | 35.62 | 0.0443 |  |
| Cardiac muscle cell development (GO:0055013) | 14.38 | 0.0443 | *SLC8A1, HNRNPU, VEGFA* |
| Regulation of cell communication by electrical coupling (GO:0010649) | 35.62 | 0.0443 | *SLC8A1, SRI* |
| Positive regulation of JUN kinase activity (GO:0043507) | 13.36 | 0.0464 | *EDN1, MAP4K2, PTPN1* |
| Response to leptin (GO:0044321) | 31.17 | 0.0498 | *EDN1, CCND1* |
| Positive regulation of cell growth involved in cardiac muscle cell development (GO:0061051) | 31.17 | 0.0498 | *EDN1, IGF1* |
| Type I pneumocyte differentiation (GO:0060509) | 31.17 | 0.0498 | *KRAS, SOX9* |
| Hepatocyte growth factor receptor signaling pathway (GO:0048012) | 31.17 | 0.0498 | *MET, NRP1* |
| Post-translational protein targeting to membrane, translocation (GO:0031204) | 31.17 | 0.0498 | *SEC61A1, SEC62* |

Bioinformatic prediction of miR-1 putative human targets was performed with TargetScan, miRTarBase and miRDB. The annotation “GO Biological Process” from GeneCodis was used to identify enriched pathways. The table shows biological processes of miR-1 putative target genes, that appeared in the three prediction programs used and reported an adjusted *p*-value < 0.05 in GeneCodis.

**Supplementary Table 2.** Primers designed for qPCR mRNA expression analysis.

| Gene Name | Reverse Primer |  | Forward Primer |
| --- | --- | --- | --- |
| *36B4* (*RPLP0*) | TGCATCAGTACCCCATTCTATCAT |  | AGGCAGATGGATCAGCCAAGA |
| *ACTB* | ACTCCTGCTTGCTGATCCAC |  | GATCATTGCTCCTCCTGAGC |
| *ACOX1* | TTCCAGGCGGGCATGA |  | TCTTCACTTGGGCATGTTCCT |
| *ADRB3* | GCATCACGAGAAGAGGAAGG |  | GCCTTCGCCTCCAACATG |
| *ATGL* (*PNPLA2*) | CTCCAGCAAGCAGATGGTGA |  | GGGAGAAGATCACGTCCTGG |
| *CIDEA* | CGTTAAGGCAGCCGATGAA |  | GCGAGAGTCACCTTCGACTTG |
| *COL1A1* | CCGCCATACTCGAACTGGAA |  | ACCTGCGTGTACCCCACTCA |
| *CPT1M* (*CHKB-CPT1B*) | GAGCAGCACCCCAATCAC |  | AACTCCATAGCCATCATCTGCT |
| *FABP4* | CAACGTCCCTTGGCTTATGCT |  | TGTGCAGAAATGGGATGGAAA |
| *FASN* | GTAGGACCCCGTGGAATGTCA |  | TGAACTCCTTGGCGGAAGAGA |
| *HSL* (*LIPE*) | GGTTCTGTGTGATCCGCTCAA |  | GCACTACAAACGCAACGAGACA |
| *PLIN1* | GATGGGAACGCTGATGCTGTT |  | ACCCCCCTGAAAAGATTGCTT |
| *PPARG2* | ATCAGTGAAGGAATCGCTTTCTG |  | CAAACCCCTATTCCATGCTGTT |
| *UCP1* | CCAGGATCCAAGTCGCAAGA |  | GTGTGCCCAACTGTGCAATG |

Primer Express Software (Perkin Elmer and Analytical Sciences, Boston, MA, USA; <http://www.perkinelmer.com>) was used to design forward and reverse oligonucleotide sequences. Abbreviations: *RPLP0* (Ribosomal Protein Lateral Stalk Subunit P0), *ACOX1* (Acyl-CoA Oxidase 1), *ACTB* (Actin Beta), *ADRB3* (Adrenoceptor Beta 3), *ATGL* (Adipose Triglyceride Lipase), *CIDEA* (Cell Death Inducing DFFA Like Effector A), *COL1A1* (Collagen Type I Alpha 1 Chain), *CPT1M* (Carnitine palmitoyltransferase I), *FABP4* (Fatty Acid Binding Protein 4), FASN (Fatty Acid Synthase), HSL (Hormone-sensitive lipase), *PLIN1* (Perilipin 1), *PPARG* (Peroxisome Proliferator-Activated Receptor Gamma), *UCP1* (Uncoupling Protein 1).

**Supplementary Table 3.** Predesigned primers used for qPCR mRNA expression analysis.

| Gene Name | Assay ID | RefSeq |
| --- | --- | --- |
| HIF3A | Hs.PT.58.3256189 | NM_022462(4) |
| MAPKAPK2 | Hs.PT.58.2443418 | NM_004759(2) |
| PTK9 | Hs.PT.58.18764315 | NM_002822(4) |
| RXRA | Hs.PT.58.3784663 | NM_002957(1) |

Oligonucleotide sequences were obtained from Integrated DNA Technologies (IDT; Coralville, IA, USA). Abbreviations: ID (identification), *HIF3A* (Hypoxia Inducible Factor 3 Subunit Alpha), *MAPKAPK2* (MAPK Activated Protein Kinase 2), *PTK9* (Protein Tyrosine Kinase 9), RefSeq (Reference Sequence), RXRA (Retinoid X Receptor Alpha).

**Supplementary Table 4.** Primers designed for qPCR mitochondrial DNA quantification analysis.

| Gene Name |  | Reverse Primer |  | Forward Primer |  |
| --- | --- | --- | --- | --- | --- |
| *LPL* |  | TTCTGGATTCCAATGCTTCGA |  | CGAGTCGTCTTTCTCCTGATGAT |  |
| *NADHdS1* |  | GAGCGATGGTGAGAGCTAAGGT |  | CCCTAAAACCCGCCACATCT |  |

Primer Express Software (Perkin Elmer and Analytical Sciences, Boston, MA, USA; <http://www.perkinelmer.com>) was used to design forward and reverse oligonucleotide sequences. Abbreviations: *LPL* (Lipoprotein Lipase), *NADHdS1* (NADH dehydrogenase Subunit 1).

**Supplementary Table 5.** Characteristics of hMADs transfection. Volumes indicated correspond to one well. Abbreviations: lipo (lipofectamine), mito (mitochondrial).

| Cell type | Assay | Plate format | 10 µM miRNA (µl) | Lipo  (µl) | Opti-MEM  (µl) | Cell Medium (µl) | Cell number |
| --- | --- | --- | --- | --- | --- | --- | --- |
| hMADS | Gene expression assays | 12-well | 1.50 | 4.50 | 150.0 | 450 | 25,0000 |
|  | Western Blot  Mito DNA quantification | 6-well | 3.00 | 9.00 | 300.0 | 900 | 50,000 |


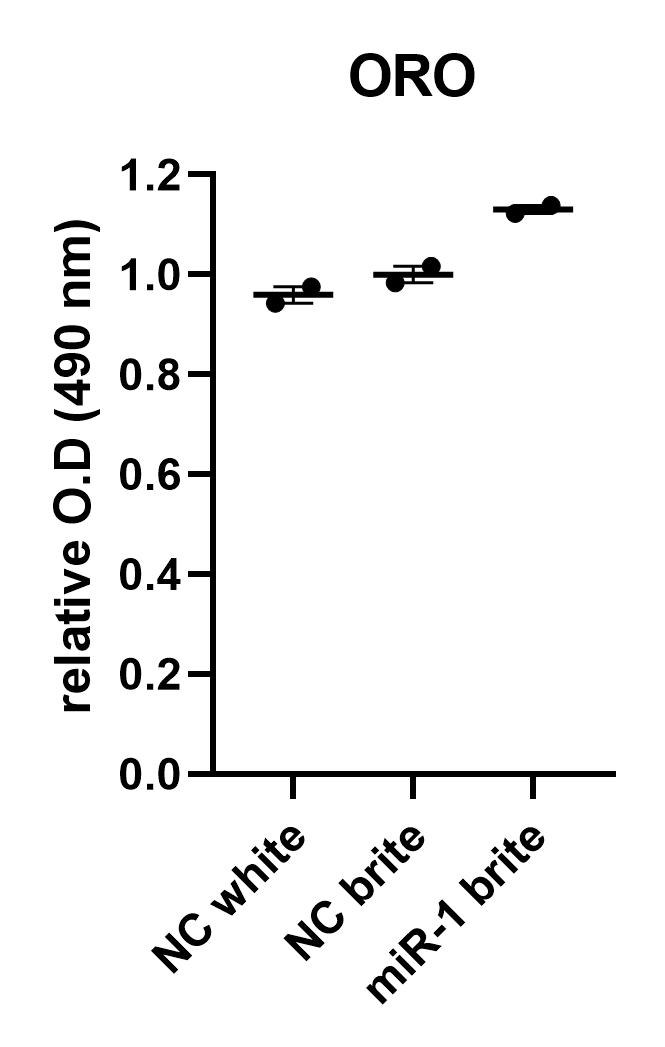


**Supplementary Figure 1**. Evaluation of Oil Red O staining in human adipocytes. hMADS cells were subjected to differentiation at day 0, transfected at day 10-12 with 25 nM of hsa-miR-1 or a scramble sequence (negative control), and stimulated to undergo browning between day 14 and 18. Oil Red O staining was quantified by dissolving stained adipocytes with isopropanol and measuring absorbance at 490 nm with a spectrophotometer. The results depicted are relative optical density (O. D.) of absorbance and expressed as mean ± standard error of the mean (SEM) (n=1, in replicate). Abbreviations: NC (negative control), mitoDNA (mitochondrial DNA).

**Supplementary Figure 2**. Evaluation of miR-1 impact on mitochondriogenesis in brown-like adipocytes. hMADS cells were subjected to differentiation at day 0, transfected at day 10-12 with 25 nM of hsa-miR-1 or a scramble sequence (negative control), and stimulated to undergo browning between day 14 and 18. DNA levels of the mitochondrial gene *NADHdS1* (NADH dehydrogenase Subunit 1) were quantified at day 18 of differentiation by qPCR in white and brite adipocytes and normalized with the nuclear gene *LPL* (Lipoprotein Lipase). The results depicted are the mitochondria DNA quantity mean (comparisons made with negative control brite using the 2−^ΔΔCt^ method) ± standard error of the mean (SEM) (n=2), and significance was determined with Student two-tailed t-test when compared to NC brite. *p*-value: * p<0.05. Abbreviations: NC (negative control), mitoDNA (mitochondrial DNA).
